# Social Media Impact of the Food and Drug Administration's Drug Safety Communication Messaging About Zolpidem: Mixed-Methods Analysis

**DOI:** 10.2196/publichealth.7823

**Published:** 2018-01-05

**Authors:** Michael S Sinha, Clark C Freifeld, John S Brownstein, Macarius M Donneyong, Paula Rausch, Brian M Lappin, Esther H Zhou, Gerald J Dal Pan, Ajinkya M Pawar, Thomas J Hwang, Jerry Avorn, Aaron S Kesselheim

**Affiliations:** ^1^ Program On Regulation, Therapeutics, And Law Division of Pharmacoepidemiology and Pharmacoeconomics, Department of Medicine Brigham and Women's Hospital and Harvard Medical School Boston, MA United States; ^2^ College of Computer and Information Science Northeastern University Boston, MA United States; ^3^ Computational Epidemiology Group Boston Children's Hospital Boston, MA United States; ^4^ Health Services Management and Policy College of Public Health The Ohio State University Columbus, OH United States; ^5^ Food and Drug Administration Silver Spring, MD United States

**Keywords:** Food and Drug Administration, drug safety communications, surveillance, epidemiology, social media, Twitter, Facebook, Google Trends

## Abstract

**Background:**

The Food and Drug Administration (FDA) issues drug safety communications (DSCs) to health care professionals, patients, and the public when safety issues emerge related to FDA-approved drug products. These safety messages are disseminated through social media to ensure broad uptake.

**Objective:**

The objective of this study was to assess the social media dissemination of 2 DSCs released in 2013 for the sleep aid zolpidem.

**Methods:**

We used the MedWatcher Social program and the DataSift historic query tool to aggregate Twitter and Facebook posts from October 1, 2012 through August 31, 2013, a period beginning approximately 3 months before the first DSC and ending 3 months after the second. Posts were categorized as (1) junk, (2) mention, and (3) adverse event (AE) based on a score between –0.2 (completely unrelated) to 1 (perfectly related). We also looked at Google Trends data and Wikipedia edits for the same time period. Google Trends search volume is scaled on a range of 0 to 100 and includes “Related queries” during the relevant time periods. An interrupted time series (ITS) analysis assessed the impact of DSCs on the counts of posts with specific mention of zolpidem-containing products. Chow tests for known structural breaks were conducted on data from Twitter, Facebook, and Google Trends. Finally, Wikipedia edits were pulled from the website’s editorial history, which lists all revisions to a given page and the editor’s identity.

**Results:**

In total, 174,286 Twitter posts and 59,641 Facebook posts met entry criteria. Of those, 16.63% (28,989/174,286) of Twitter posts and 25.91% (15,453/59,641) of Facebook posts were labeled as junk and excluded. AEs and mentions represented 9.21% (16,051/174,286) and 74.16% (129,246/174,286) of Twitter posts and 5.11% (3,050/59,641) and 68.98% (41,138/59,641) of Facebook posts, respectively. Total daily counts of posts about zolpidem-containing products increased on Twitter and Facebook on the day of the first DSC; Google searches increased on the week of the first DSC. ITS analyses demonstrated variability but pointed to an increase in interest around the first DSC. Chow tests were significant (*P*<.0001) for both DSCs on Facebook and Twitter, but only the first DSC on Google Trends. Wikipedia edits occurred soon after each DSC release, citing news articles rather than the DSC itself and presenting content that needed subsequent revisions for accuracy.

**Conclusions:**

Social media offers challenges and opportunities for dissemination of the DSC messages. The FDA could consider strategies for more actively disseminating DSC safety information through social media platforms, particularly when announcements require updating. The FDA may also benefit from directly contributing content to websites like Wikipedia that are frequently accessed for drug-related information.

## Introduction

When the Food and Drug Administration (FDA) learns about new or emerging adverse drug reactions potentially caused by an FDA-regulated product, the agency may issue a Drug Safety Communication (DSC) to health care professionals, patients, and the public to help them make more informed medication-related decisions [1,2]. These safety issues can include new side effect profiles [3], dosing adjustments [4], previously unknown adverse events (AEs) [5], including increased risk of death [6], drug-drug interactions [7], and differential responses between patient subgroups [8].

The FDA disseminates safety messages about prescription and over-the-counter (OTC) drugs through online media outlets, as well as through numerous other platforms, to make information available where people will see it. The Internet is a primary source of medical information for a growing number of people, particularly through searches on Google and from resources such as Wikipedia [9]. Health care providers also frequently use online resources [10]. As news and information have become portable through mobile phones and other devices, social media platforms such as Twitter and Facebook have grown as a means of sharing personal experiences and opinions [11]. Given that 70% of Facebook users and 38% of Twitter users access the platforms daily, and that in 2012 approximately 28% of Facebook profiles [12] and 88% of Twitter feeds [13] were public, these platforms enable users to routinely and broadly share personal experiences with medical products. Social media can potentially have promising applications for public health and drug safety surveillance [14-17], though the potential for inaccurate information, length constraints, and other drawbacks may limit this utility.

The FDA issued a total of 233 DSCs between 2010 and 2016 [18]. In 2013, 2 DSCs were issued related to the sedative/hypnotic zolpidem (Ambien), which was first approved in 1992 to treat insomnia. On January 10, 2013, the FDA released a DSC that warned, in part, about the risk of next-morning impairment and recommended lower starting doses for zolpidem, particularly in women [19]. A follow-up DSC was released on May 14, 2013 providing updated information on the specific FDA-approved label changes for the affected zolpidem products, along with a recommendation to avoid driving the day after using extended-release versions of the product [20]. As part of its dissemination strategy for all DSCs, FDA actively communicated the key message from each zolpidem DSC via some FDA social media accounts.

To assess online activity following the release of DSC messaging for zolpidem, we assessed trends in daily mentions on Twitter and Facebook, searches in Google, and edits to the zolpidem Wikipedia page. Our goal was to determine the extent to which safety messages were shared through social media and the content and wording of user posts about zolpidem, including how they may have changed following the release of the information in the DSCs.

## Methods

### Facebook and Twitter Posts

To collect Facebook and Twitter posts, we used MedWatcher Social, a media monitoring program developed by Epidemico and the Computational Epidemiology Group at Boston Children’s Hospital and Harvard Medical School, based on technology from HealthMap [21]. Using the DataSift historic query tool, we collected historical Twitter and Facebook posts ranging from October 1, 2012 through August 31, 2013, a time period that allowed us to follow activity from approximately 3 months before the first DSC (DSC1) through 3 months after the second DSC (DSC2). We executed searches for English-language posts using the following queries: ambien, ambian, zolpidem.

The DataSift tool delivered the results in files in a standardized JavaScript Object Notation (JSON) format; the post content and date were then extracted and stored in a database system for ease of automated processing and manual review. Each post was then placed into 1 of the following 3 categories using an automated content classification algorithm: (1) junk, (2) mention, and (3) adverse event (AE). The classification algorithm is a machine learning system based on a Fisher-Robinson classifier and has previously been described in detail [17,22]. For a given post, it outputs a score signifying its likelihood of being relevant to discussion of an AE, ranging from –0.2 (completely unrelated) to 1 (perfectly related). The training set for the algorithm consists of over 411,000 manually-labeled historical dataset posts categorized as either AE or non-AE.

Posts with scores below 0.02 are tagged as junk, those with scores greater than or equal to 0.02 and less than 0.7 are labeled as mention, and those scoring greater than or equal to 0.7 are marked as AE. AE posts were intended to capture negative outcomes attributed to the product. Mention posts represented legitimate mentions of the product, but not attributing any adverse outcome to it. The junk category was intended to collect and filter advertising, promotional, automated, spam, or otherwise irrelevant content. Repeated discussions of a single event were de-duplicated if they were posted twice within a 1-hour period with no or minimal changes to the text. Unexpected spikes in the Facebook and Twitter data were further examined for fidelity. Through this process, we found that spikes in counts of Facebook or Twitter postings on all other days besides the day of either DSC were largely due to misclassification of “spam storms” (large volumes of posts relating to advertisement, promotional, automated, or otherwise irrelevant content) as relevant postings. For example, we manually verified a spam storm resulting in 8243 Facebook posts on June 28, 2013, and an additional 505 posts the following day. To normalize the findings, June 28 data were replaced by the mean number of posts from June 21 to 27, and June 29 data were replaced by the mean number of posts from June 30 to July 6.

Separately, we collected posts from FDA Twitter and Facebook accounts relating to the 2 DSCs. Four accounts were identified as possible sources of data: 1 on Facebook (@FDA, the main FDA page) and 3 on Twitter (@US_FDA, @FDA_Drug_Info, and @FDAMedWatch). Relevant posts can be found in Multimedia Appendix 1. Though FDA posts could not be specifically identified in the dataset, manual verification of posts containing language similar to the FDA’s were often identified and counted as junk in the MedWatcher Social analysis.

### Google and Wikipedia Data

Google Trends data are comprised of an unbiased sample of Google search data, with each value representing a random sample of searches for a given time period [23]. Data are scaled on a range of 0 to 100, with 100 representing the maximum number of searches during the relevant time period. For shorter periods, daily results are available, but for longer time windows, data are reported weekly. For a given search, a score of 100 represented the day or week with the greatest number of individual queries for the drug over the selected time frame. The “Related queries” section of the Google trends output page provided information as to similar searches that were “Rising” in the relevant time period [24]. Any search with a greater than 5000% increase in search frequency is defined by Google Trends as a Breakout search. We searched for the term “ambien” in the same time interval as our Twitter and Facebook data (October 1, 2012 to August 31, 2013), obtaining weekly data points, with additional Google Trends searches reporting daily results during the months of January 2013 and May 2013 to focus in on the relevant DSC time periods. Given the likelihood that the average person would not remember or search for the trade name zolpidem, we elected to query the most popularized brand name.

Wikipedia edits were pulled from the editorial history of the page for “Zolpidem” (searches for Ambien are redirected to this page) [25]. The editorial history shows when revisions were made to the page and the content of those revisions side-by-side with the original text. We queried the history for revisions made in the timeframe surrounding the 2 DSCs and manually examined the relevant content changes for completeness and accuracy. Relevant editorial changes are shown in Multimedia Appendix 1.

### Data Analysis

Data posted on Facebook and Twitter from October 2012 to August 2013 were collected and analyzed for their timing and content. We plotted the time series of historical daily Twitter and Facebook post counts. Google Trends data were also charted as a time series during the same time frame. All data analyses were conducted retrospectively using historical posts without identifiable data.

The intervals between the 3 segments, defined by DSC dates (Period 1 representing time from first data collection to DSC1, Period 2 representing time between DSC1 and DSC2, and Period 3 defined the time from DSC2 to end of data collection), varied in duration according to the outcome of interest, with the baseline trend arising from Period 1. We fitted segmented linear regression models to the ITS data to estimate the impact of each DSC. Since the structural breaks of interest were known a priori, we conducted Chow tests to assess for the presence of a structural break at the DSC times.

Analyses were conducted using Microsoft Excel and SAS (version 9.4). The study protocol was approved by the Institutional Review Board at Brigham & Women’s Hospital and FDA’s Research Involving Human Subjects Committee.

## Results

### Facebook and Twitter Posts

A total of 174,286 Twitter posts (tweets) and 59,641 Facebook posts met entry criteria, dating between October 1, 2012 and August 31, 2013. Among the tweets, 9.21% (16,051/174,286) were classified as AEs, 74.16% (129,246/174,286) as mentions, and 16.63% (28,989/174,286) as junk. Among Facebook posts, 5.11% (3050/59,641) were flagged as AEs, 68.98% (41,138/59,641) as mentions, and 25.91% (15,453/59,641) as junk. Because data were collected anonymously, it was not possible to ascertain the total number of unique individuals who generated these posts.

**Figure 1:**
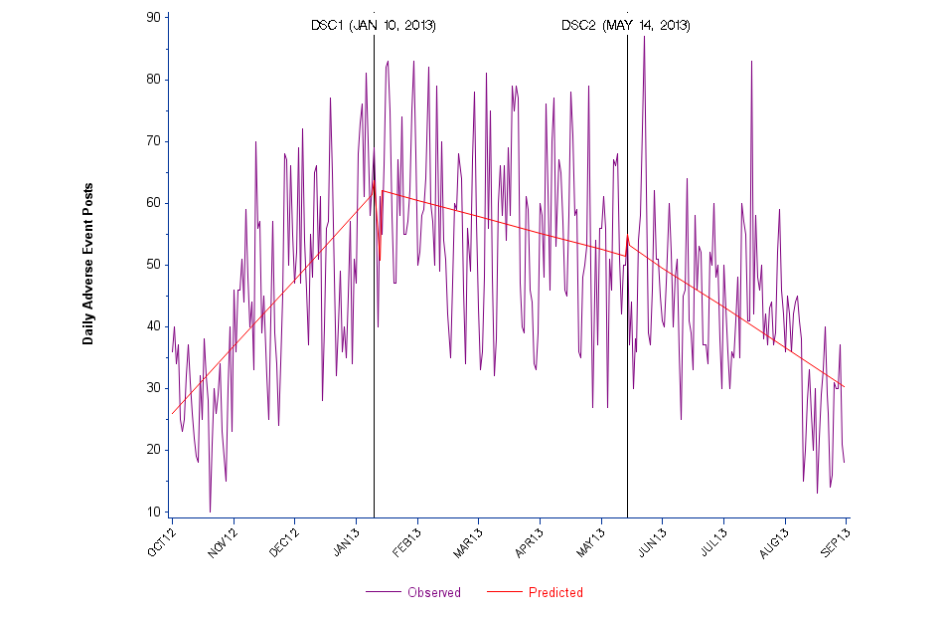
Daily Twitter adverse event posts about zolpidem (Ambien) from October 2012 to August 2013.

**Figure 2:**
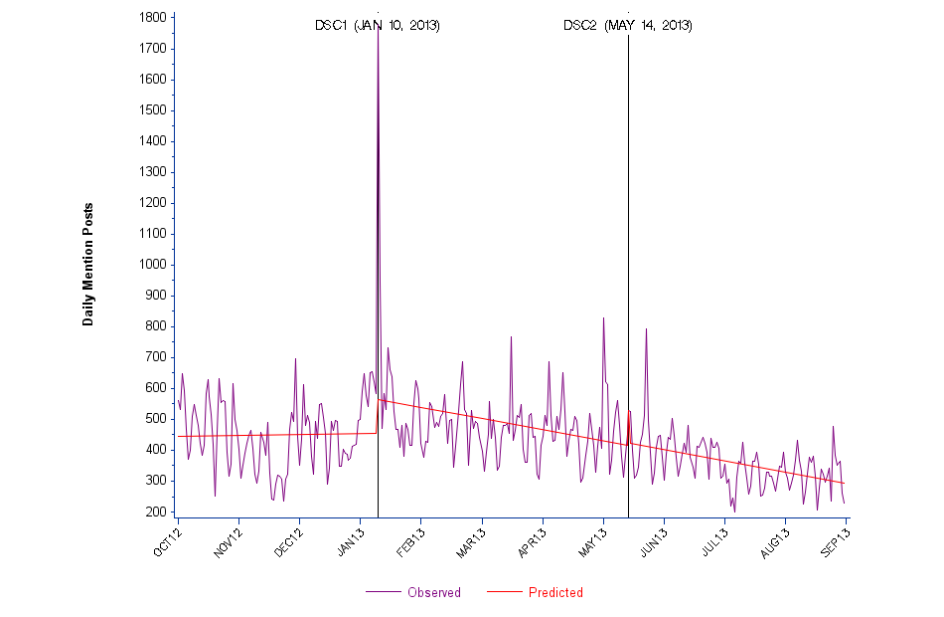
Daily Twitter mention posts about zolpidem (Ambien) from October 2012 to August 2013.

**Figure 3:**
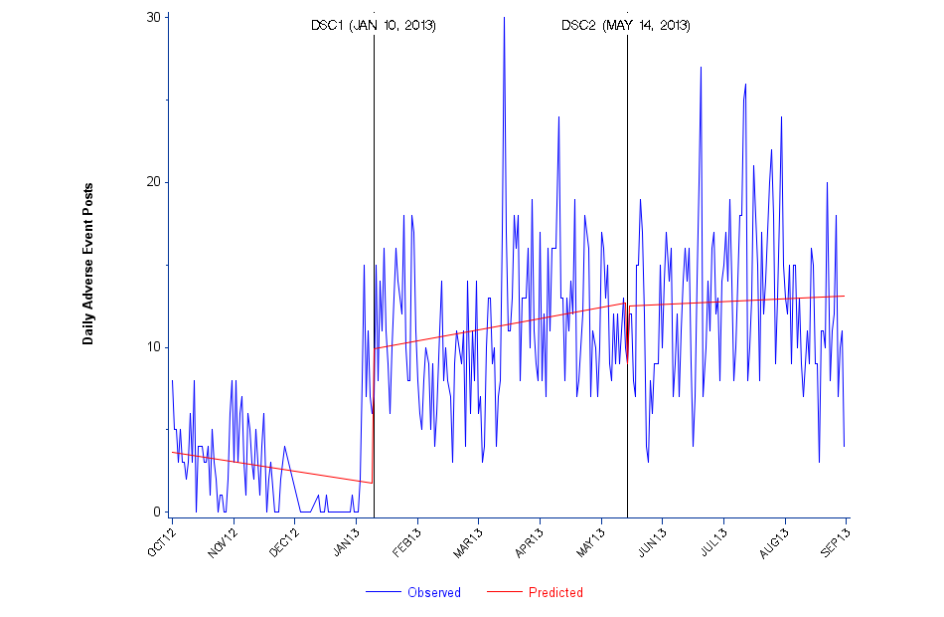
Daily Facebook adverse event posts about zolpidem (Ambien) from October 2012 to August 2013. DSC: drug safety communication.

**Figure 4:**
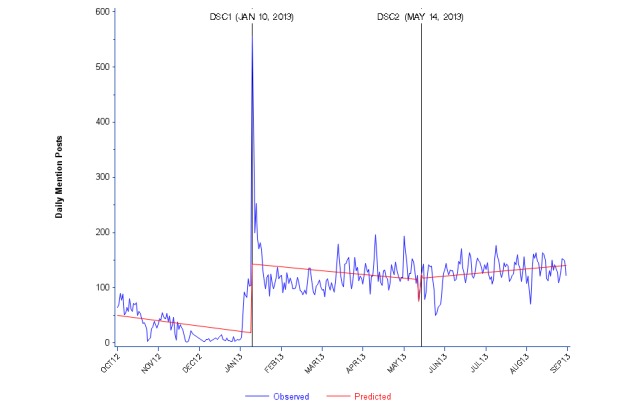
Daily Facebook mention posts about zolpidem (Ambien) from October 2012 to August 2013. DSC: drug safety communication.

Time series plots of daily counts of posts for each category of posts are presented together with their predicted regression lines (Figures 1-4). For data outputs corresponding to each segmented linear regression model, see Multimedia Appendix 2. Overall, we observed substantial variability in the daily counts of posts for both social media sources. Chow tests demonstrated statistical significance (*P*<.0001) for Twitter and Facebook posts at both DSC1 and DSC2. Overall counts of daily AE posts on Twitter varied from less than 10 to more than 80. ITS effect estimates were significant for all 3 periods. For the baseline Period 1, we observed a steady increase in numbers of posts. Periods 2 and 3 were marked by a decreasing trend over time after DSC1 and DSC2 (Figure 1 and Multimedia Appendix 2).

For Twitter daily mention posts, we observed a spike at DSC1 but not at DSC2. This large and statistically significant (*P*=.01) increase in posts at DSC1 was followed by a significant declining trend (*P*=.01) during Period 2. We did not observe additional changes in Period 3 compared with Period 2 (Figure 2 and Multimedia Appendix 2).

There were few daily posts in Facebook tagged as AEs, with a strikingly low number (approaching zero) in the 3 months leading up to DSC1. We observed a significant (*P*<.0001) positive change at DSC1, but no significant change at DSC2 (Figure 3 and Multimedia Appendix 2).

There were also few Facebook posts tagged as mentions in the 3 months leading up to DSC1, although they then spiked significantly (*P*<.0001) at DSC1, similar to the Twitter findings. No significant change was observed at DSC2 (Figure 4 and Multimedia Appendix 2). Daily posts in Facebook tagged as AEs (increased about 6 per day) and mentions (increased about 100 per day) then plateaued and were sustained at a higher level after DSC1.

### FDA Accounts

Communications arising from FDA on the day of DSC1 release included 4 tweets sent from the @FDA_Drug_Info account (retweeted a collective 71 times), 1 tweet from the @FDAMedWatch account (retweeted 24 times), and 1 tweet from the @US_FDA account (retweeted 16 times). There was a single Facebook post published that day that had 61 shares.

For DSC2, no Facebook posts were made by FDA and no tweets were sent from the main @US_FDA Twitter account. Three tweets were sent from the @FDA_Drug_Info account with a collective 37 retweets. The @FDAMedWatch account tweeted a generic message related to all the recent prescribing changes FDA had made recently to 48 products, which did not mention zolpidem by name. It was retweeted 3 times, but only a single reply to the original post referenced the drug: “check out revisions… esp Ambien”. That post was not retweeted.

Each FDA tweet linked to a different internal Web page posted on the FDA website. For example, the @FDA_Drug_Info DSC1 tweets linked to the original DSC, a Spanish version of the DSC, and a related consumer article and press release. The DSC2 tweets linked to the DSC, its Spanish version, and an MP3 podcast addressing the DSC (see Multimedia Appendix 1).

### Google Searches

**Figure 5:**
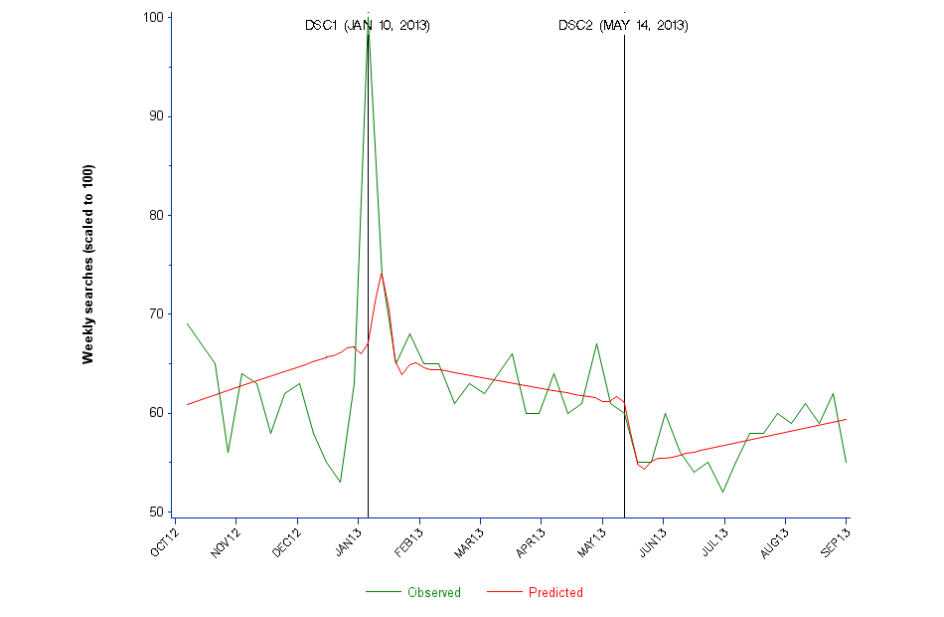
Weekly searches for zolpidem/Ambien on Google (scaled to 100) from October 2012 to August 2013.

The Google Trends graph for US-based Web searches for “Ambien” between October 1, 2012 and August 31, 2013 [26] reached a peak of 100 during the week of January 6 to January 12, 2013, which includes the release of DSC1 (Figure 5 and Multimedia Appendix 2). ITS was not significant for searches at DSC1 and DSC2 or within Periods 1 to 3, but Chow tests demonstrated statistical significance for Google at DSC1 but not DSC2.

The Google Trends plot mirrors the Facebook and Twitter Mentions data in 2 important ways: each has a visible peak at DSC1, but lacks any visible change at DSC2. Related queries rising in frequency over this 11-month time period include “ambien fda warning” (+1, 100%), “fda ambien” (+500%), “ambien dosage women” (+450%), “ambien warning” (+450%), “ambien and women” (+130%), and “ambien news” (+50%). In the graphs for each of these related queries, the peak centers around DSC1 but low search volume for these terms precludes further analysis. Search frequency for these multiple-word searches was lower than searches for “ambien”.

When focusing more closely on the dates around the 2 zolpidem DSCs, the January 2013 graph of Google Trends Web searches [26] identified a peak around January 10 to January 11, 2013, returning to baseline within a few days. The volume of news searches showed a similar trend. Related queries for “ambien fda”, “ambien warning”, and “ambien fda warning” were classified as Breakout (+ >5000%), and “ambien news” (+850%) also increased significantly during the month of January. In May 2013 [26], the peak search for “ambien” occurred on May 1, steadily declining through the month. There was a brief uptick of searches on May 14 to May 15, the day after the DSC was issued, but it did not exceed search frequency from May 1. Related queries in May did not pertain to the FDA or drug warnings and instead focused on Ambien more generally: “ambien side effects”, “ambien dosage,” and “ambien generic”. None were rising or breakout, meaning that search frequency of these terms did not change to an appreciable extent during May 2013.

### Wikipedia Changes

An addition was made to the last paragraph of the opening section of the zolpidem Wikipedia page on January 10, 2013 to reflect some information included in DSC1. Citing a CBS News article, the page notes:

On January 10, 2013, the FDA announced it is requiring the manufacturer of Ambien and Zolpimist to cut the recommended dosage in half for women after laboratory studies showed that the medications can leave patients drowsy in the morning and at risk for car accidents.

It was edited again on April 30, 2013 to add the following information, the first sentence of which was included in DSC1:

The FDA recommended that manufacturers extend the new dosage cuts to men as well, who process the drug at a faster rate. However, the reasons why men and women catabolize the drugs at different rates is still unknown.

No additional citation was provided for this addendum.

Wikipedia page edits for zolpidem on May 15, 2013 included information from DSC2:

In May 2013, the FDA approved label changes specifying new dosage recommendations for Zolpidem products because of concerns regarding next-morning impairment.

The reference cited for this addition was an article on the Lawyers and Settlements website. The DSC language has since been moved up to the second paragraph of the article, but still does not contain a reference to the FDA DSC. As of January 3, 2018, the citations for these 2013 edits remain unchanged.

## Discussion

### Principal Findings

In this analysis, we examined uptake by various social media outlets of 2 FDA DSCs related to the sedative/hypnotic zolpidem, as well as associated changes in Google searches and updates to Wikipedia. These communication pathways can lead to more widespread dissemination of the messaging in DSCs [9]. We observed a similar spike of Facebook and Twitter daily mentions, as well as weekly Google searches at DSC1; but the sharp increase in engagement was not sustained. We also found a significant increase in the number of daily AEs posted on Facebook after DSC1. Daily posts on Facebook tagged as AEs and as mentions both plateaued and were then sustained at a higher level after DSC1. By contrast, DSC2 largely failed to gain additional traction, reflected by no visible increase in Facebook and Twitter posts or Google searches at the time of DSC2.

This study builds on previous work looking at social media and pharmacovigilance in the United States [27-34] and Europe [35,36], in addition to a growing body of work on FDA DSC messaging [37] by systematically evaluating the social media impact of DSCs. There are a number of possible explanations for the differential effects of the 2 DSCs in social media. For example, DSC1 was accompanied by a press release and had more FDA-originated messaging on Facebook and Twitter as compared to DSC2. In addition, users of Twitter and Facebook may have perceived DSC2 as clarifying DSC1 rather than providing new information. Indeed, the FDA’s web page for DSC2 notes: “This update is in follow-up to the FDA Drug Safety Communication issued on 1/10/2013”. Finally, zolpidem was not mentioned by name in FDA social messaging at the time of DSC2, which may have muted the immediacy of the public health information.

Facebook and Twitter reflect public conversations with peers, while Google Trends data reflect anonymous user searches for information, but the results were generally consistent. Google Trends “Related queries” for Ambien in January 2013 included the words “FDA” and “warning”, suggesting that users were searching Google for information pertaining to DSC1; however, searches for Ambien steadily declined through the month of May 2013. DSC2, therefore, stimulated less investigatory online activity and interpersonal communication.

Although Wikipedia was updated close to the original release of the DSCs, the editors did not cite the original DSCs from the FDA webpage and the January 2013 edit of zolpidem’s Wikipedia page was incomplete. It took until April 30, 2013 for the Wikipedia page to be updated with an additional detail from DSC1. The information included on DSC2 was added to the Wikipedia page quickly as well. Given that informational sites like Wikipedia are commonly accessed by the lay public for information on drugs and that anyone can edit the content, the FDA could consider a plan to formally update the pages for appropriate content at the time a DSC is released and to ensure the continued accuracy of the information over time.

The FDA has a wide following on Facebook (528,000 likes as of September 2017) and Twitter (@US_FDA has 175,000 followers as of September 2017, @FDA_Drug_Info has 231,000 followers, and @FDAMedWatch has 38,700 followers). Social media communications should continue to be part of future public drug safety communications and consideration should be given to expanding their use in the context of DSC-related messaging. But what is the optimal amount and duration of social media necessary to maximize public health benefits? Social media may provide a timely, singular update about changes to important prescribing information, but social media discussions are generally short-lived, while information on the proper use of a medicine needs to be available consistently. For example, if users of zolpidem are engaging on social media to learn about recent updates about their medicine, the transient social media interest in the zolpidem DSC is not likely to benefit future users of the medicine, who may find other sources (such as a Google search, Wikipedia, or the FDA website) more valuable. Future strategies for using social media should be based on a more detailed understanding of user profiles and preferences. FDA may develop multiple approaches to disseminating DSC messages, including posting the same information multiple times, because a single post may often be overlooked by followers.

### Limitations

Some observed data could not be explained, such as the drop in Facebook posts to near-zero in December 2012. Coupled with lower daily post counts compared to Twitter, significant findings from Facebook data must be interpreted in this light. We could only observe public Facebook accounts and public posts from non-public accounts, and daily counts of Facebook posts were considerably lower than that of Twitter, so we may be underestimating the Facebook impact of the DSCs.

There is also the possibility of misclassification by the MedWatcher Social program, which may not be the optimal tool for the FDA to utilize when tracking DSC dissemination over time. The tool is designed to identify posts from individual users related to AEs, which may differ from the FDA’s needs with regard to DSC content dissemination, including through news outlets. For instance, several posts excluded as junk by MedWatcher Social were from news sources reporting on the 2 DSCs. As a result, public interest in each DSC may have been underestimated.

As compared to Facebook and Twitter, Google Trends search data are aggregated, anonymous, and lack the privacy restrictions that may have precluded access to certain relevant Facebook and Twitter posts. However, the granularity of available Google Trends data (with weekly, rather than daily, data points) may have limited statistical power, though the general trend resembled that of mention posts on the other platforms. We did not cover all major search engines or other Web-based and mobile technologies to allow for a fuller view across major social channels. This particular social media study was conducted as a subset of a multimodal analysis of FDA DSC messages using zolpidem as an example [37]; therefore, we only evaluated social media content related to zolpidem DSCs. DSCs for other medical products are likely to have differential impacts and outcomes. In addition, the zolpidem DSCs were posted in 2013. The social media environment has changed significantly since then. Future studies including DSCs from multiple medications and issued more recently will provide comprehensive insight.

### Conclusion

Our study of drug safety information dissemination through Twitter, Facebook, Google searches, and Wikipedia following the release of 2 DSCs providing key changes in prescribing recommendations related to zolpidem found substantial but short-lived social media uptake of only 1 of the 2 information releases. Outcomes from this case should be compared with uptake observed around other DSC messages and other drug safety-related content to help the FDA expand dissemination of these important messages and provide the greatest public health impact.

## Appendix

Multimedia Appendix 1 Food and Drug Administration (FDA) social media posts about Ambien.

Multimedia Appendix 2 Interrupted time series (ITS) and Chow tests.

## Abbreviations

AE: adverse event
DSC: drug safety communication
FDA: Food and Drug Administration
ITS: interrupted time series
OTC: over-the-counter

## References

[1] Dal Pan GJ (2012). Communicating the risks of medicines: time to move forward. Med Care.

[2] Ishiguro C, Hall M, Neyarapally GA, Dal Pan GJ (2012). Post-market drug safety evidence sources: an analysis of FDA drug safety communications. Pharmacoepidemiol Drug Saf.

[3] Lasser KE, Allen PD, Woolhandler SJ, Himmelstein DU, Wolfe SM, Bor DH (2002). Timing of new black box warnings and withdrawals for prescription medications. JAMA.

[4] (2011). Food and Drug Administration.

[5] Avorn J (2007). In defense of pharmacoepidemiology--embracing the yin and yang of drug research. N Engl J Med.

[6] Van Assche G, Van Ranst M, Sciot R, Dubois B, Vermeire S, Noman M, Verbeeck J, Geboes K, Robberecht W, Rutgeerts P (2005). Progressive multifocal leukoencephalopathy after natalizumab therapy for Crohn's disease. N Engl J Med.

[7] (2011). Food and Drug Administration.

[8] Giner L, Nichols CM, Zalsman G, Oquendo MA (2005). Selective serotonin reuptake inhibitors and the risk for suicidality in adolescents: an update. Int J Adolesc Med Health.

[9] Hwang TJ, Bourgeois FT, Seeger JD (2014). Drug safety in the digital age. N Engl J Med.

[10] Egle JP, Smeenge DM, Kassem KM, Mittal VK (2015). The Internet School of Medicine: use of electronic resources by medical trainees and the reliability of those resources. J Surg Educ.

[11] Duggan M (2015). Pew Research Center: Internet & Technology.

[12] (2012). Consumer Reports.

[13] Bosker B (2012). The Huffington Post.

[14] Huesch MD, Galstyan A, Ong MK, Doctor JN (2016). Using social media, online social networks, and internet search as platforms for public health interventions: a pilot study. Health Serv Res.

[15] Eysenbach G (2009). Infodemiology and infoveillance: framework for an emerging set of public health informatics methods to analyze search, communication and publication behavior on the Internet. J Med Internet Res.

[16] Brownstein JS, Freifeld CC, Madoff LC (2009). Digital disease detection--harnessing the Web for public health surveillance. N Engl J Med.

[17] Pierce CE, Bouri K, Pamer C, Proestel S, Rodriguez HW, Van Le H, Freifeld CC, Brownstein JS, Walderhaug M, Edwards IR, Dasgupta N (2017). Evaluation of Facebook and Twitter monitoring to detect safety signals for medical products: an analysis of recent FDA safety alerts. Drug Saf.

[18] (2016). Food and Drug Administration.

[19] (2013). Food and Drug Administration.

[20] (2013). Food and Drug Administration.

[21] MedWatcher.

[22] Robinson G (2003). A statistical approach to the spam program. Linux J.

[23] (2017). Google Search Trends Help.

[24] (2017). Google Search Trends Help.

[25] (2017). Wikipedia.

[26] Google Trends.

[27] Liu J, Zhao S, Zhang X (2016). An ensemble method for extracting adverse drug events from social media. Artif Intell Med.

[28] Liu X, Chen H (2015). A research framework for pharmacovigilance in health social media: identification and evaluation of patient adverse drug event reports. J Biomed Inform.

[29] Lardon J, Abdellaoui R, Bellet F, Asfari H, Souvignet J, Texier N, Jaulent M, Beyens M, Burgun A, Bousquet C (2015). Adverse drug reaction identification and extraction in social media: a scoping review. J Med Internet Res.

[30] Sarker A, Ginn R, Nikfarjam A, O'Connor K, Smith K, Jayaraman S, Upadhaya T, Gonzalez G (2015). Utilizing social media data for pharmacovigilance: a review. J Biomed Inform.

[31] Powell GE, Seifert HA, Reblin T, Burstein PJ, Blowers J, Menius JA, Painter JL, Thomas M, Pierce CE, Rodriguez HW, Brownstein JS, Freifeld CC, Bell HG, Dasgupta N (2016). Social media listening for routine post-marketing safety surveillance. Drug Saf.

[32] Bahk CY, Goshgarian M, Donahue K, Freifeld CC, Menone CM, Pierce CE, Rodriguez H, Brownstein JS, Furberg R, Dasgupta N (2015). Increasing patient engagement in pharmacovigilance through online community outreach and mobile reporting applications: an analysis of adverse event reporting for the Essure Device in the US. Pharmaceut Med.

[33] Duh MS, Cremieux P, Audenrode MV, Vekeman F, Karner P, Zhang H, Greenberg P (2016). Can social media data lead to earlier detection of drug-related adverse events?. Pharmacoepidemiol Drug Saf.

[34] Korkontzelos I, Nikfarjam A, Shardlow M, Sarker A, Ananiadou S, Gonzalez GH (2016). Analysis of the effect of sentiment analysis on extracting adverse drug reactions from tweets and forum posts. J Biomed Inform.

[35] Coloma PM, Becker B, Sturkenboom MCJM, van Mulligen EM, Kors JA (2015). Evaluating social media networks in medicines safety surveillance: two case studies. Drug Saf.

[36] Bagheri H, Lacroix I, Guitton E, Damase-Michel C, Montastruc J (2016). Cyberpharmacovigilance: what is the usefulness of the social networks in pharmacovigilance?. Therapie.

[37] Kesselheim AS, Campbell EG, Schneeweiss S, Rausch P, Lappin BM, Zhou EH, Seeger JD, Brownstein JS, Woloshin S, Schwartz LM, Toomey T, Dal Pan GJ, Avorn J (2015). Methodological approaches to evaluate the impact of FDA drug safety communications. Drug Saf.

