# Supplementary material for: Social Media Impact of the Food and Drug Administration's Drug Safety Communication Messaging About Zolpidem: Mixed-Methods Analysis
Source: JMIR Public Health Surveill. 2018 Jan 5;4(1):e1. doi: 10.2196/publichealth.7823 (PMC5775485; doi:10.2196/publichealth.7823)
Supplement: Multimedia Appendix 1 [file publichealth_v4i1e1_app1.pdf]

APPENDIX 1:

Uptake of FDA Drug Safety Communication Messages about Zolpidem in Social Media

Sinha MS, Freifeld CC, Brownstein JS, Donneyong M, Rausch P, Lappin BM, Zhou EH, Dal Pan GJ, Pawar AM, Hwang TJ, Avorn J, Kesselheim AS

FDA Accounts (Twitter and Facebook, as of 1/31/2017):

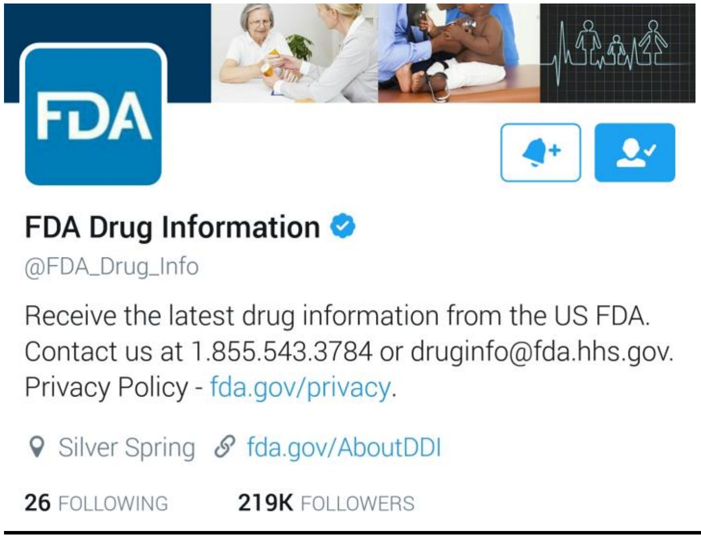

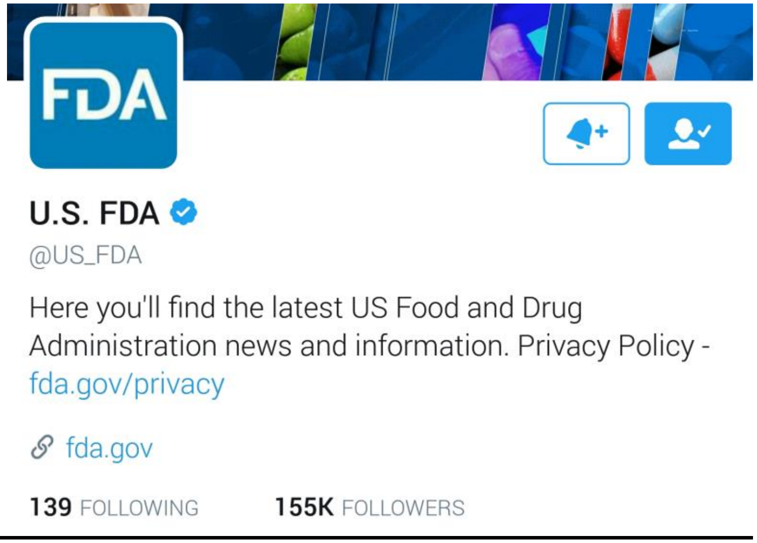

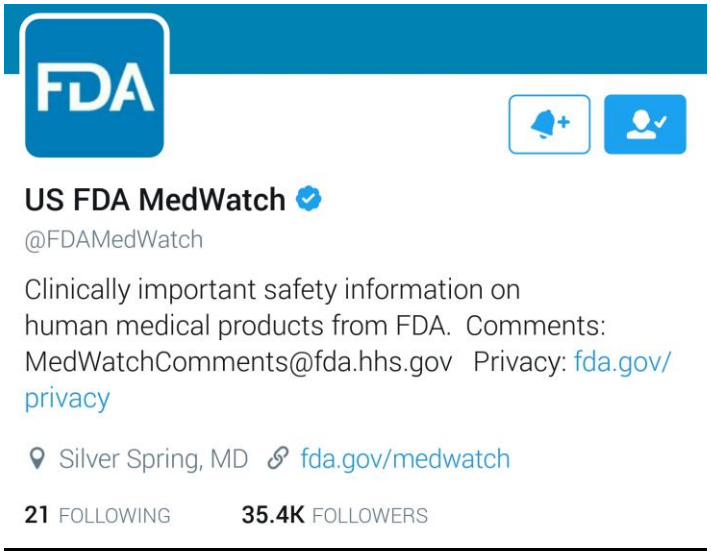

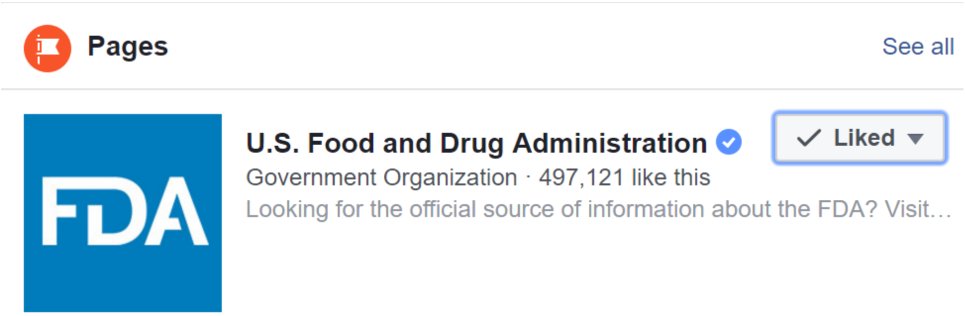

Twitter Posts for DSCs:

Twitter @US_FDA DSC1: https://twitter.com/US_FDA/status/289392030013071360
(Links to FDA News Release)

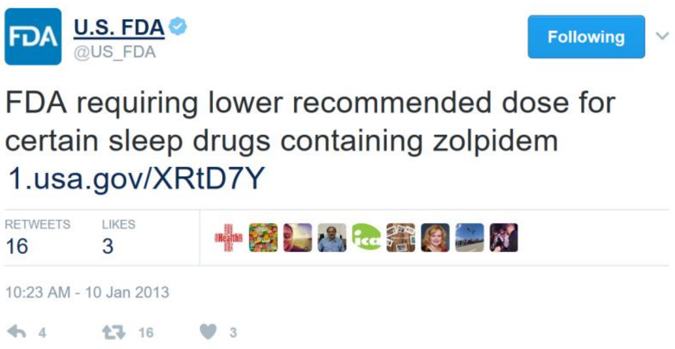

Twitter @FDAMedWatch DSC1:
https://twitter.com/FDAMedWatch/status/289390432490766336 (Links to MedWatch safety information)

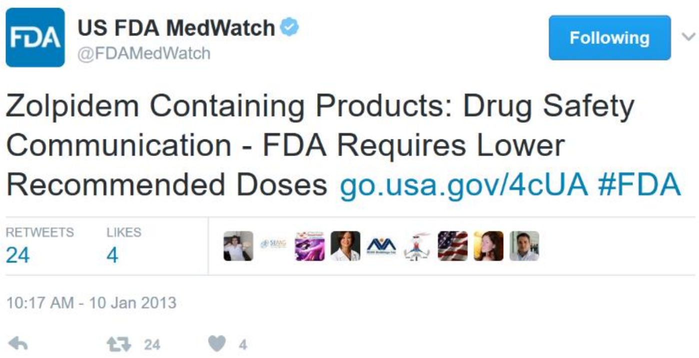

Twitter @FDA_Drug_Info DSC1:
https://twitter.com/FDA_Drug_Info/status/289403721027297281 (Links to DSC)
https://twitter.com/FDA_Drug_Info/status/289402157625651201 (Links to News Release)
https://twitter.com/FDA_Drug_Info/status/289457255303241728 (Links to Consumer Page)
https://twitter.com/FDA_Drug_Info/status/289438247774482432 (Links to DSC in Spanish)

FDA Drug Information
@FDA_Drug_Info
Following

#DSC: Risk of next-morning impairment, #FDA requires lower rec doses for certain drugs containing zolpidem: 1.usa.gov/U7QdVj.

RETWEETS 12 LIKE 1

11:09 AM - 10 Jan 2013

12 1

FDA Drug Information
@FDA_Drug_Info
Following

#FDA requiring lower recommended dose for certain sleep drugs containing zolpidem: 1.usa.gov/11jqL4y

RETWEETS 18 LIKES 5

11:03 AM - 10 Jan 2013

18 5

FDA Drug Information
@FDA_Drug_Info
Following

#FDA wants dose lowered for #sleep #drugs with zolpidem, like #Ambien -- they could impair #driving. See go.usa.gov/YjhH

RETWEETS 31 LIKES 5

2:42 PM - 10 Jan 2013

4 31 5

FDA Drug Information
@FDA_Drug_Info
Following

En Espanol: Risk of next-morning impairment, FDA requires lower rec doses for certain Rxs containing zolpidem 1.usa.gov/U8fuyL

RETWEETS 10 LIKE 1

1:27 PM - 10 Jan 2013

10 1

Twitter @FDA_Drug_Info DSC2:
https://twitter.com/FDA_Drug_Info/status/334309502058893314 (Links to DSC)
https://twitter.com/FDA_Drug_Info/status/335092983059853314 (Links to MP3 audio of Podcast)
https://twitter.com/FDA_Drug_Info/status/335430188361998336 (Links to DSC in Spanish)

FDA Drug Information
@FDA_Drug_Info
Following

Drug Safety: Label changes for zolpidem products&new dosing recommendation for the day after Ambien CR use 1.usa.gov/15JgnXq

RETWEETS 26 LIKES 7

10:09 AM - 14 May 2013

1 26 7

FDA Drug Information
@FDA_Drug_Info
Following

En #Espanol: #FDA approves label changes for #zolpidem products 1.usa.gov/16EgGSS

RETWEETS 6 LIKES 4

12:22 PM - 17 May 2013

1 6 4

FDA Drug Information
@FDA_Drug_Info
Following

#Podcast: Label changes for #zolpidem products&new dosing recommendation for the day after #Ambien CR use. 1.usa.gov/17AkViw

RETWEETS 5 LIKES 5

2:02 PM - 16 May 2013

1 5 5

Twitter @FDAMedWatch DSC2:
https://twitter.com/FDAMedWatch/status/334829078639153152 (Links to MedWatch April 2013 Spreadsheet)

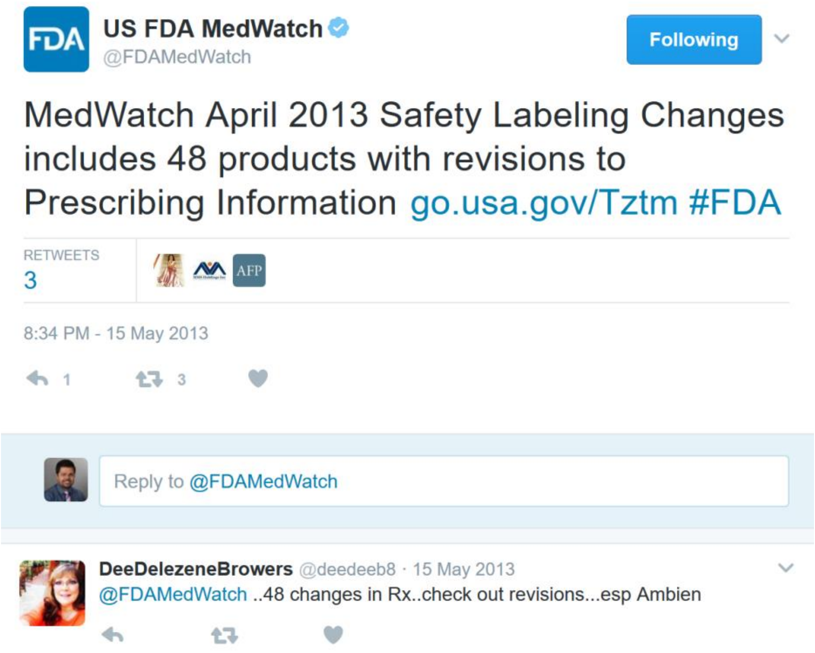

US FDA MedWatch
@FDAMedWatch

Following

MedWatch April 2013 Safety Labeling Changes includes 48 products with revisions to Prescribing Information go.usa.gov/Tztm #FDA

RETWEETS
3

AFP

8:34 PM - 15 May 2013

1 3

Reply to @FDAMedWatch

DeeDelezeneBrowers @deedeeb8 · 15 May 2013
@FDAMedWatch ..48 changes in Rx..check out revisions...esp Ambien

FDA Posts for DSCs:

Facebook FDA DSC1:
https://www.facebook.com/plugins/post.php?href=https%3A%2F%2Fwww.facebook.com%2FFDA%2Fposts%2F489802544403848
(Links to FDA Consumer page)

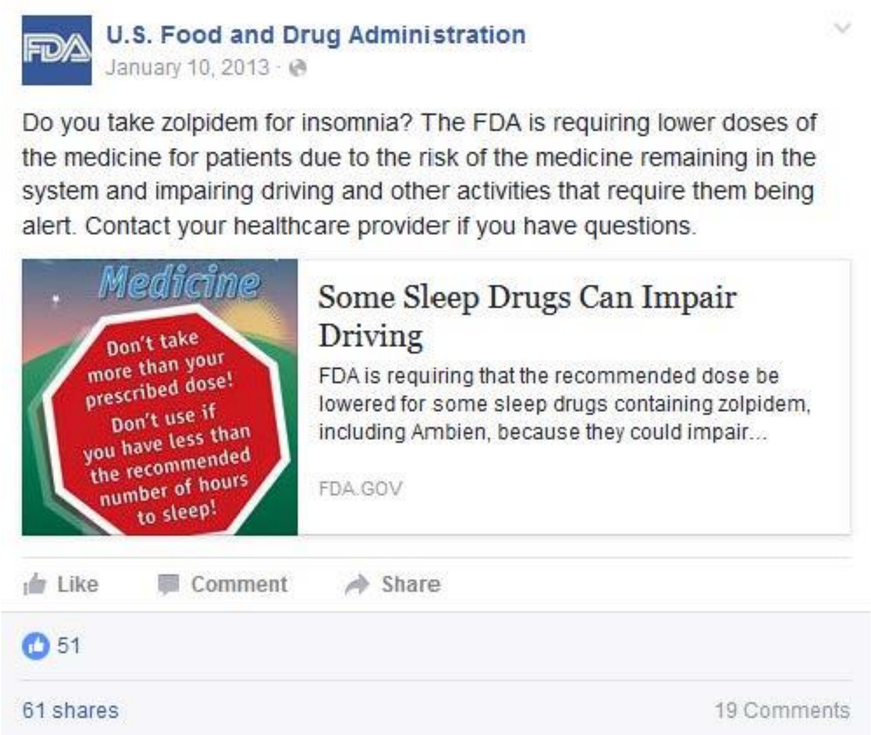

Wikipedia Edits for DSCs:

https://en.wikipedia.org/w/index.php?title=Zolpidem&type=revision&diff=532412924&oldid=532089163
https://en.wikipedia.org/w/index.php?title=Zolpidem&type=revision&diff=555264437&oldid=552878249

Zolpidem: Difference between revisions

From Wikipedia, the free encyclopedia

Revision as of 02:25, 9 January 2013 (edit)<br>1canuckbuck (talk | contribs)<br>← Previous edit	Revision as of 19:38, 10 January 2013 (edit) (undo)<br>Kennvido (talk | contribs)<br>*(FDA requirement)*<br>Next edit →
**Line 63:**	**Line 63:**
The patent in the United States on zolpidem was held by the French pharmaceutical corporation [[Sanofi-Aventis]].<ref name="US_4382938">{{Ref patent | country = US | number = 4382938 | title = Imidazo[1,2-a] pyridine derivatives and their application as pharmaceuticals | pubdate = 1983-05-10 | gdate = 1984-07-17 | inventor = Kaplan J-P, George P | assign1 = Synthelabo }}</ref> On April 23, 2007, the [[Food and Drug Administration (United States)|U.S. Food and Drug Administration]] (FDA) approved 13 generic versions of zolpidem tartrate.<ref name="urlFDA Approves First Generic Versions of Ambien (Zolpidem Tartrate) for the Treatment of Insomnia">{{cite web |url=http://www.fda.gov/NewsEvents/Newsroom/PressAnnouncements/2007/ucm108897.htm |title=FDA Approves First Generic Versions of Ambien (Zolpidem Tartrate) for the Treatment of Insomnia |work= |accessdate=2010-01-24}}</ref> Zolpidem is available from several generic manufacturers in the UK, as a generic from [[Sandoz]] in South Africa and [[Teva Pharmaceutical Industries|TEVA]] in Israel, as well as from other manufacturers such as [[Ratiopharm]] (Germany).	The patent in the United States on zolpidem was held by the French pharmaceutical corporation [[Sanofi-Aventis]].<ref name="US_4382938">{{Ref patent | country = US | number = 4382938 | title = Imidazo[1,2-a] pyridine derivatives and their application as pharmaceuticals | pubdate = 1983-05-10 | gdate = 1984-07-17 | inventor = Kaplan J-P, George P | assign1 = Synthelabo }}</ref> On April 23, 2007, the [[Food and Drug Administration (United States)|U.S. Food and Drug Administration]] (FDA) approved 13 generic versions of zolpidem tartrate.<ref name="urlFDA Approves First Generic Versions of Ambien (Zolpidem Tartrate) for the Treatment of Insomnia">{{cite web |url=http://www.fda.gov/NewsEvents/Newsroom/PressAnnouncements/2007/ucm108897.htm |title=FDA Approves First Generic Versions of Ambien (Zolpidem Tartrate) for the Treatment of Insomnia |work= |accessdate=2010-01-24}}</ref> Zolpidem is available from several generic manufacturers in the UK, as a generic from [[Sandoz]] in South Africa and [[Teva Pharmaceutical Industries|TEVA]] in Israel, as well as from other manufacturers such as [[Ratiopharm]] (Germany).
	+
	+ On January 10, 2013, the [[Food and Drug Administration]] announced it is requiring the manufacturer of Ambien and Zolpimist to cut the recommended dosage in half for women after laboratory studies showed that the medicines can leave patients drowsy in the morning and at risk for car accidents.<ref>{{cite web|title=FDA tells drugmakers to lower doses for Ambien, other sleeping pills|url=http://www.cbsnews.com/8301-204_162-57563277/fda-tells-drugmakers-to-lower-doses-for-ambien-other-sleeping-pills/|publisher=CBS News|accessdate=10 January 2013}}</ref>
==Medical uses==	==Medical uses==

Zolpidem: Difference between revisions

From Wikipedia, the free encyclopedia

Revision as of 12:50, 30 April 2013 (edit)
110.175.63.51 (talk)
(→Adverse effects: *Poor software development is too specific*)
← Previous edit

Revision as of 20:19, 15 May 2013 (edit) (undo)
64.134.229.187 (talk)
Next edit →

Line 65:

The United States patent for zolpidem was held by the French pharmaceutical corporation [[Sanofi-Aventis]].<ref name="US_4382938">{{Ref patent | country = US | number = 4382938 | title = Imidazo[1,2-a] pyridine derivatives and their application as pharmaceuticals | pubdate = 1983-05-10 | gdate = 1984-07-17 | inventor = Kaplan J-P, George P | assign1 = Synthelabo }}</ref> On April 23, 2007, the [[Food and Drug Administration (United States)|U.S. Food and Drug Administration]] (FDA) approved 13 generic versions of zolpidem tartrate.<ref name="urlFDA Approves First Generic Versions of Ambien (Zolpidem Tartrate) for the Treatment of Insomnia">{{cite web |url=http://www.fda.gov/NewsEvents/Newsroom/PressAnnouncements/2007/ucm108897.htm |title=FDA Approves First Generic Versions of Ambien (Zolpidem Tartrate) for the Treatment of Insomnia |work= |accessdate=2010-01-24}}</ref> Zolpidem is available from several generic manufacturers in the UK, as a generic from [[Sandoz]] in South Africa and [[Teva Pharmaceutical Industries|TEVA]] in Israel, as well as from other manufacturers such as [[Ratiopharm]] (Germany).

−

On January 10, 2013, the [[Food and Drug Administration]] announced it is requiring the manufacturer of Ambien and Zolpimist to cut the recommended dosage for women in half, after laboratory studies showed that the medicines can leave patients drowsy in the morning and at risk for car accidents.The FDA recommended that manufacturers extend the new dosage cuts to men as well, who process the drug at a faster rate. However, the reasons why men and women catabolize the drugs at different rates is still unknown. <ref>{{cite web|title=FDA tells drugmakers to lower doses for Ambien, other sleeping pills|url=http://www.cbsnews.com/8301-204_162-57563277/fda-tells-drugmakers-to-lower-doses-for-ambien-other-sleeping-pills/|publisher=CBS News|accessdate=10 January 2013}}</ref>

Line 65:

The United States patent for zolpidem was held by the French pharmaceutical corporation [[Sanofi-Aventis]].<ref name="US_4382938">{{Ref patent | country = US | number = 4382938 | title = Imidazo[1,2-a] pyridine derivatives and their application as pharmaceuticals | pubdate = 1983-05-10 | gdate = 1984-07-17 | inventor = Kaplan J-P, George P | assign1 = Synthelabo }}</ref> On April 23, 2007, the [[Food and Drug Administration (United States)|U.S. Food and Drug Administration]] (FDA) approved 13 generic versions of zolpidem tartrate.<ref name="urlFDA Approves First Generic Versions of Ambien (Zolpidem Tartrate) for the Treatment of Insomnia">{{cite web |url=http://www.fda.gov/NewsEvents/Newsroom/PressAnnouncements/2007/ucm108897.htm |title=FDA Approves First Generic Versions of Ambien (Zolpidem Tartrate) for the Treatment of Insomnia |work= |accessdate=2010-01-24}}</ref> Zolpidem is available from several generic manufacturers in the UK, as a generic from [[Sandoz]] in South Africa and [[Teva Pharmaceutical Industries|TEVA]] in Israel, as well as from other manufacturers such as [[Ratiopharm]] (Germany).

+

On January 10, 2013, the [[Food and Drug Administration]] announced it is requiring the manufacturer of Ambien and Zolpimist to cut the recommended dosage for women in half, after laboratory studies showed that the medicines can leave patients drowsy in the morning and at risk for car accidents.The FDA recommended that manufacturers extend the new dosage cuts to men as well, who process the drug at a faster rate. However, the reasons why men and women catabolize the drugs at different rates is still unknown. <ref>{{cite web|title=FDA tells drugmakers to lower doses for Ambien, other sleeping pills|url=http://www.cbsnews.com/8301-204_162-57563277/fda-tells-drugmakers-to-lower-doses-for-ambien-other-sleeping-pills/|publisher=CBS News|accessdate=10 January 2013}}</ref> **In May 2013, the FDA approved label changes specifying new dosage recommendations for Zolpidem products because of concerns regarding next-morning impairment. <ref>http://www.lawyersandsettlements.com/articles/personal_injury/Ambien-FDA-Dosing-changes-18721.html#.UZPs8St35cI</ref>**
