# Supplementary material for: Social Media Impact of the Food and Drug Administration's Drug Safety Communication Messaging About Zolpidem: Mixed-Methods Analysis
Source: JMIR Public Health Surveill. 2018 Jan 5;4(1):e1. doi: 10.2196/publichealth.7823 (PMC5775485; doi:10.2196/publichealth.7823)
Supplement: Multimedia Appendix 2 [file publichealth_v4i1e1_app2.pdf]

APPENDIX 2:

Uptake of FDA Drug Safety Communication Messages about Zolpidem in Social Media

Sinha MS, Freifeld CC, Brownstein JS, Donneyong M, Rausch P, Lappin BM, Zhou EH, Dal Pan GJ, Pawar AM, Hwang TJ, Avorn J, Kesselheim AS

Twitter

Table 1: Daily Twitter Adverse Event posts about zolpidem/Ambien, Oct 2012-August 2013

Interrupted time trend of ***zolpidem-containing products***: R^2 = **0.39**	Effect Estimate	Standard Error	P Value
Intercept	26.02	2.42	<.0001
Baseline trend	0.34	0.04	<.0001
Change in counts of posts at DSC1	0.02	3.29	0.99
Change in counts of posts per day after DSC1	-0.43	0.05	<.0001
Change in counts of posts at DSC2	2.14	3.25	0.51
Change in counts of posts per day after DSC2	-0.12	0.05	0.01
Regression model: Counts of zolpidem posts = $\beta_0 + \beta_1 time_t + \beta_2(\textit{post DSC1})_t + \beta_3(\textit{time post DSC1})_t + \beta_4(\textit{post DSC2})_t + \beta_5(\textit{time post DSC2})_t + e_t$			

**Chow test**		
Break Point	F Value	Pr > F
1/10/2013	100.05	<.0001
5/14/2013	57.72	<.0001

Table 2: Daily Twitter Mention posts about zolpidem/Ambien, Oct 2012-August 2013

Interrupted time trend of ***zolpidem-containing products***: R^2 = **0.24**	Effect Estimate	Standard Error	P Value
Intercept	445.88	23.50	<.0001
Baseline trend	0.10	0.40	0.80
Change in counts of posts at DSC1	109.48	31.38	0.01
Change in counts of posts per day after DSC1	-1.30	0.50	0.01
Change in counts of posts at DSC2	12.09	30.73	0.69
Change in counts of posts per day after DSC2	-0.05	0.46	0.91
Regression model: Counts of zolpidem posts = $\beta_0 + \beta_1 time_t + \beta_2(\textit{post DSC1})_t + \beta_3(\textit{time post DSC1})_t + \beta_4(\textit{post DSC2})_t + \beta_5(\textit{time post DSC2})_t + e_t$			

**Chow test**		
Break Point	F Value	Pr > F
1/10/2013	27.9	<.0001
5/14/2013	14.82	<.0001

Facebook

Table 3: Daily Facebook Adverse Event posts about zolpidem/Ambien

Interrupted time trend of ***zolpidem-containing products***: R^2 = **0.51**	Effect Estimate	Standard Error	P Value
Intercept	3.63	0.89	<.0001
Baseline trend	-0.02	0.02	0.24
Change in counts of posts at DSC1	8.10	1.16	<.0001
Change in counts of posts per day after DSC1	0.04	0.02	0.04
Change in counts of posts at DSC2	-0.28	1.10	0.80
Change in counts of posts per day after DSC2	-0.02	0.02	0.36
Regression model: Counts of zolpidem posts = $\beta_0 + \beta_1 time_t + \beta_2(post\ DSC1)_t + \beta_3(time\ post\ DSC1)_t + \beta_4(post\ DSC2)_t + \beta_5(time\ post\ DSC2)_t + e_t$			

**Chow test**		
Break Point	F Value	Pr > F
1/10/2013	41.28	<.0001
5/14/2013	14.21	<.0001

Table 4: Daily Facebook Mention posts about zolpidem/Ambien

Interrupted time trend of ***zolpidem-containing products***: R^2 = **0.56**	Effect Estimate	Standard Error	P Value
Intercept	51.04	8.19	<.0001
Baseline trend	-0.38	0.16	0.02
Change in counts of posts at DSC1	126.07	10.69	<.0001
Change in counts of posts per day after DSC1	0.14	0.19	0.44
Change in counts of posts at DSC2	-0.26	10.09	0.98
Change in counts of posts per day after DSC2	0.48	0.15	0.01
Regression model: Counts of zolpidem posts = $\beta_0 + \beta_1 time_t + \beta_2(post\ DSC1)_t + \beta_3(time\ post\ DSC1)_t + \beta_4(post\ DSC2)_t + \beta_5(time\ post\ DSC2)_t + e_t$			

**Chow test**		
Break Point	F Value	Pr > F
1/10/2013	87.74	<.0001
5/14/2013	13.2	<.0001

Google

Table 5: Weekly Searches for zolpidem/Ambien on Google, scaled to 100

Interrupted time trend of ***zolpidem-containing products***: R^2 = **0.27**	Effect Estimate	Standard Error	P Value
Intercept	59.55	3.59	<.0001
Baseline trend	0.64	0.39	0.11
Change in weekly searches at DSC1	-3.74	4.67	0.43
Change in weekly searches after DSC1	-0.90	0.51	0.09
Change in weekly searches at DSC2	-6.43	4.63	0.17
Change in weekly searches after DSC2	0.56	0.49	0.25
Regression model: Counts of zolpidem posts = $\beta_0 + \beta_1 time_t + \beta_2(\textit{post DSC1})_t + \beta_3(\textit{time post DSC1})_t + \beta_4(\textit{post DSC2})_t + \beta_5(\textit{time post DSC2})_t + e_t$			

**Chow test**		
Break Point	F Value	Pr > F
1/10/2013	8.44	0.0008
5/14/2013	1.9	0.1615
